# Neuronal Loss after Stroke Due to Microglial Phagocytosis of Stressed Neurons

**DOI:** 10.3390/ijms222413442

**Published:** 2021-12-14

**Authors:** Guy C. Brown

**Affiliations:** Department of Biochemistry, University of Cambridge, Cambridge CB2 1QW, UK; gcb3@cam.ac.uk

**Keywords:** stroke, cell death, neuronal death, delayed neuronal death, selective neuronal loss, secondary neurodegeneration, phagocytosis, microglia, phagoptosis, ischemia

## Abstract

After stroke, there is a rapid necrosis of all cells in the infarct, followed by a delayed loss of neurons both in brain areas surrounding the infarct, known as ‘selective neuronal loss’, and in brain areas remote from, but connected to, the infarct, known as ‘secondary neurodegeneration’. Here we review evidence indicating that this delayed loss of neurons after stroke is mediated by the microglial phagocytosis of stressed neurons. After a stroke, neurons are stressed by ongoing ischemia, excitotoxicity and/or inflammation and are known to: (i) release “find-me” signals such as ATP, (ii) expose “eat-me” signals such as phosphatidylserine, and (iii) bind to opsonins, such as complement components C1q and C3b, inducing microglia to phagocytose such neurons. Blocking these factors on neurons, or their phagocytic receptors on microglia, can prevent delayed neuronal loss and behavioral deficits in rodent models of ischemic stroke. Phagocytic receptors on microglia may be attractive treatment targets to prevent delayed neuronal loss after stroke due to the microglial phagocytosis of stressed neurons.

## 1. Introduction

Stroke is an acute reduction in blood flow within the brain and a major cause of mortality and morbidity throughout the world [1]. Stroke rapidly kills neurons in the brain areas of lowest blood flow, resulting in an infarct of necrotic brain tissue; but stroke also induces the delayed death and loss of neurons in brain areas surrounding the infarct (the penumbra) or brain areas distant but neuronally connected to the infarct [2,3,4,5,6]. Here we review the evidence that this delayed neuronal death is due to the microglial phagocytosis of neurons and, therefore, may be prevented by blocking this phagocytosis.

## 2. Types of Neuronal Death after Stroke

**Figure 1:**
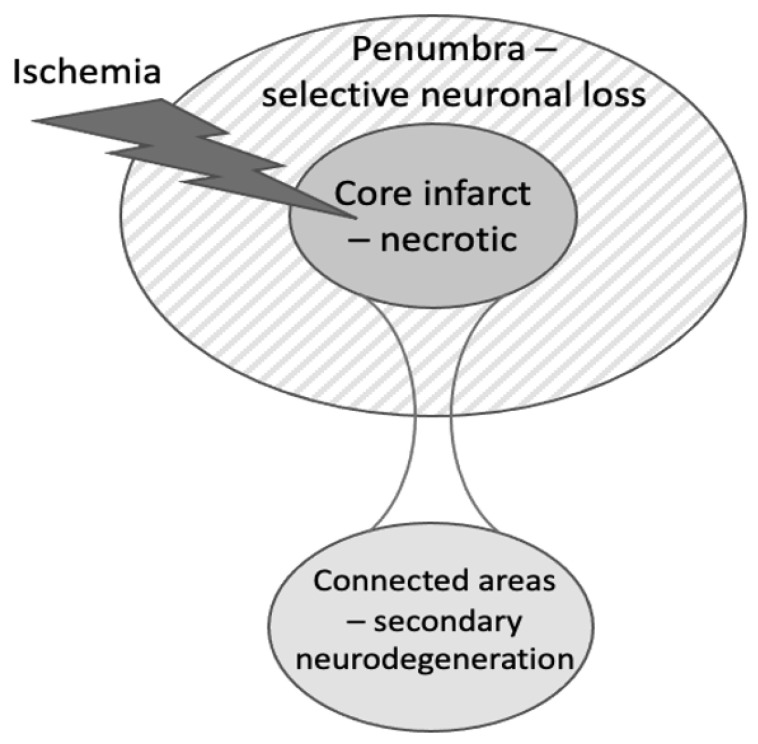
Types of cell death induced in different brain areas after stroke. In the brain area of deepest ischemia, virtually all cells die rapidly by necrosis, resulting in the infarct. In the ischemic penumbra surrounding the infarct, there is a delayed and selective loss of neurons. In brain areas distant from the infarct but neuronally connected to the infarct, there is a delayed loss of neurons, known as secondary neurodegeneration.

Stoke rapidly induces necrosis of all cells within the infarct; however, two types/locations of delayed neuronal death after brain ischemia have also been identified (Figure 1). (1) ‘Selective neuronal loss’ may occur in peri-infact areas 1–7 days after transient middle cerebral artery occlusion (MCAO) in rodent striatum (and to a lesser extent in the cortex), but may increase over the following weeks [4,7,8,9,10,11,12,13]. (2) ‘Secondary neurodegeneration’ of the thalamus may occur 0.5–12 months after cortical stroke [2,3,14]. There may also be hippocampal neurodegeneration secondary to cortical infarcts [15] and neurodegeneration in the midbrain secondary to infarcts in the striatum [16,17,18]. ‘Selective neuronal loss’ refers to the selective loss of neurons in contrast to the loss of all cell types in the infarct [4,7,8,9]. Selective neuronal loss is alternatively referred to as ‘selective neuronal death’ or ‘selective neuronal necrosis’ [9,10]. It is possible that some selective neuronal loss in the peri-infarct area may be caused by the degeneration of neuronal connections to the infarct area. In addition, very transient focal ischemia can cause selective neuronal loss without an infarct. For example, 30 min of MCAO in rats induced selective neuronal loss in the striatum without any infarct, 5 days after the ischemia, and accompanied by dramatic activation of microglia [19]. In addition, transient global ischemia in rodents and cardiac arrest in humans may induce delayed neuronal death (after 1–3 days) in the neocortex, striatum and CA1 hippocampus [4,5,6,20]. This neuronal loss after global ischemia is not necessarily relevant to stroke but may share mechanisms. Some published examples of delayed neuronal death are presented in Table 1.

Delayed neuronal death after stroke is potentially more preventable/treatable than the acute neuronal death within the infarct because: (i) the patient has time to reach a hospital and be treated over the time course of this delayed death, (ii) the brain regions with delayed neuronal death are perfused with blood, and therefore drugs can reach the affected cells, (iii) the neurons undergoing delayed neuronal death have probably received less damage/insult than neurons within the infarct, and/or (iv) these neurons may be undergoing forms of cell death that are potentially more amenable to treatment [11,12]. Thus, it is important to understand the mechanisms by which such delayed neuronal death occurs.

Neurons can die in several different ways, including: intrinsic and extrinsic apoptosis, oncosis, necroptosis, parthanatos, ferroptosis, sarmoptosis, autophagic cell death, autosis, autolysis, paraptosis, pyroptosis, phagoptosis, mitochondrial permeability transition, aberrant cell cycle re-entry, excitotoxicity and oxytosis [21]. There is evidence that some of these mechanisms contribute to delayed neuronal death after stroke [21]. However, in this article, we will focus on phagoptosis as a cause of this delayed neuronal death. Phagoptosis is cell death resulting from phagocytosis of the cell by a phagocyte [22]. For neurons, this is usually due to stressed or damaged neurons being phagocytosed by microglia [23,24], but monocyte-derived or perivascular macrophages may contribute as phagocytes, causing, for example, damage to the blood–brain barrier [25]. In addition, excessive phagocytosis of parts of neurons (synapses, dendrites and axons) may result in secondary neuronal death [26]. It used to be thought that only dead or dying neurons are phagocytosed, but we now know synapses, dendrites, axons, neuronal precursors, newborn neurons and ‘excess’ neurons are all phagocytosed alive during development [26]. Microglial phagocytosis of neurons and neuronal parts continues in the adult brain, mediating both physiology and pathology [24,27].

## 3. Microglia and Microglial Phagocytosis of Neurons

Microglia are macrophages resident in the brain, retina and spinal cord only [28]. Microglia are the brain’s main phagocytes, i.e., cells specialized in phagocytosis, that is, the engulfment and digestion of extracellular material, including other cells [28]. In the healthy brain, microglia are highly ramified, with long, thin processes capable of phagocytosing small objects such as synapses and debris, but not capable of phagocytosing large objects, such as neurons [29]. However, when activated by inflammatory stimuli, microglia increase the expression of phagocytic receptors, opsonins and lysosomes, and the microglial processes are retracted, resulting in a large, motile cell body capable of phagocytosing neurons [29]. 

Microglial activation is closely associated with delayed neuronal loss in peri-infarct areas [4,19,30,31] but was found to slightly precede neuronal loss in a rat model of mild MCAO for 20 min, where microglial activation was not significant in the striatum at day 1 but progressively increased on days 2, 3 and 7. In contrast, neuronal loss was not significant on day 2 but increased progressively on days 3 and 7 [11]. This is consistent with (but does not show) microglial activation, causing delayed neuronal loss. However, an inhibitor of microglial activation (TRAM-34, a blocker of calcium-activated potassium channels) prevented the delayed neuronal loss and behavioral deficits induced by 15 min MCAO in hypertensive rats [12]. This suggests that microglial activation caused delayed neuronal loss. However, microglial activation can be damaging to neurons by several mechanisms other than phagocytosis [32], and certain types of microglial activation can be beneficial after stroke [33].

Microglial/macrophage phagocytosis of neurons may be beneficial in a number of ways: (i) it removes dead neurons and neuronal debris, which are pro-inflammatory and disrupt the brain, (ii) it removes dying neurons (such as apoptotic neurons) before they become necrotic and release debris, and (iii) it may remove damaged neurons that disrupt the brain, e.g., by being epileptogenic [33,34]. However, microglial/macrophage phagocytosis of neurons may also be detrimental by removing neurons that would otherwise be functional. Therefore, whether microglial/macrophage phagocytosis of neurons is net beneficial or detrimental after stroke is an empirical question that may vary with conditions. We review the evidence below as to whether inhibition of phagocytic signaling is beneficial or detrimental in stroke pathology. However, first, we review the signals that regulate the microglial phagocytosis of neurons.

## 4. Phagocytic Signaling in Stroke

How are neurons and neuronal parts phagocytosed by microglia? In general, in order for a neuron or neuronal part to be phagocytosed, it needs to: (i) release a “find-me” signal (such as ATP), that chemoattracts phagocytes (such as microglia), (ii) expose an “eat-me” signal (such as phosphatidylserine), and/or (iii) bind to an opsonin (such as MFG-E8 or complement component C1q), which normally binds to an eat-me signal on the neuron and to a phagocytic receptor on the phagocyte [35]. Healthy neurons and neuronal parts do not do the above (i–iii), and therefore are not phagocytosed, but stressed, damaged or dying neurons or neuronal parts may do one or more of the above (i–iii), potentially resulting in their phagocytosis. 

*Find-me signals* are signals released by target cells to chemoattract phagocytes, which then phagocytose the target cell [35]. Find-me signals, known to be released by neurons to chemoattract microglia/macrophages in stroke models, include the following. The nucleotides ATP and ADP chemoattract microglia via the microglial P2Y_12_ receptor [36]. P2Y_12_ receptor knockout or inhibition by clopidogrel reduced microglial migration and clustering around neurons and subsequent neuronal damage after transient brain ischemia [36]. Lysophosphatidylcholine (LPC) can chemoattract microglia via G protein-coupled receptor 132 (G2A) [37]. Fractalkine (CX3CL1) released from neurons can chemoattract microglia via the fractalkine receptor (CX3CR1) [38], and fractalkine knockout mice had less neuronal damage after transient focal ischemia [39]. Sphingosine-1-phosphate (S1P) may also chemoattract microglia, and blocking the S1P_2_ receptor prevented microglial recruitment and activation in stroke models [40]. Complement component C3a is released during a stroke and is potentially chemotactic for microglia, and a C3a receptor antagonist (SB 290157) reduced microglial recruitment and phagocytosis after ischemia in a mouse stroke model [41]. C3a receptor inhibition or knockout also reduced excessive microglial phagocytosis of intact myelin 14 days after ischemia in a mouse model [42]. 

**Figure 2:**
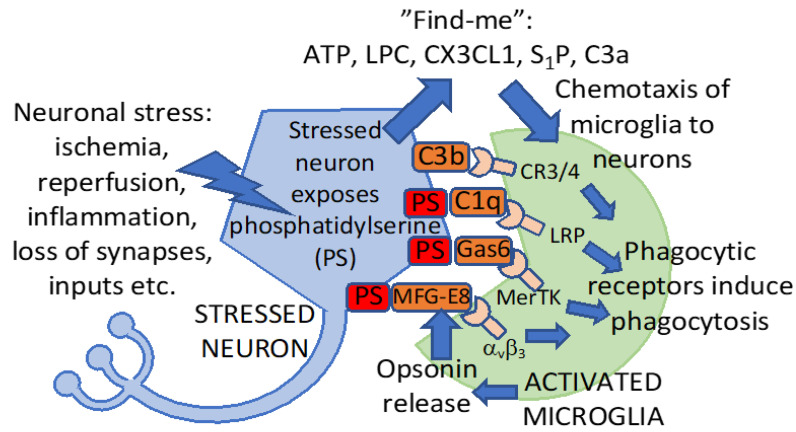
Signaling between neurons and microglia inducing phagocytosis. After a stroke, neurons may be stressed by ongoing ischemia, oxidants, inflammation, excitotoxins or the loss of synapses. Neuronal stress can induce the release of “find-me” signals and exposure of the “eat-me” signal phosphatidylserine (PS, red), which binds opsonins (brown, released by activated microglia), which then bind phagocytic receptors on microglia to induce phagocytosis. ATP, adenosine triphosphate; LPC, lysophosphatidylcholine; CX3CL1, fractalkine; S1P, sphingosine-1-phosphate; C3a, C3b, C1q: complement components 3a, 3b and 1q; Gas6, growth arrest–specific 6; MFG-E8, milk fat globule-EGF factor 8 protein; MerTK, mer tyrosine kinase; integrin αvβ3, vitronectin receptor; LRP, low density lipoprotein receptor-related protein (a phagocytic receptor for C1q); CR3, complement receptor 3; and CR4, complement receptor 4. The activation of these receptors by their opsonins bound to neurons induces microglial phagocytosis of these neurons.

*Eat-me signals* are signals displayed by a cell to induce the phagocytosis of that cell by a phagocyte [35]. The main eat-me signal driving the phagocytosis of neurons by microglia is phosphatidylserine [23]. Phosphatidylserine is a phospholipid, which is confined to the inner leaflet of the plasma membrane in healthy neurons but is permanently exposed on the outer leaflet of the plasma membrane in apoptotic or necrotic neurons [35]. However, stressed neurons can transiently expose phosphatidylserine (driven by elevated calcium or oxidants or decreased ATP), which can induce the phagocytosis of stressed neurons by microglia [23]. For example, sub-toxic levels of glutamate can induce neurons to reversibly expose phosphatidylserine on their surface, inducing their phagocytosis by microglia, and this can be prevented by blocking the exposed phosphatidylserine [43]. If microglia are present at the time that neurons expose phosphatidylserine, then the microglia phagocytose such neurons. However, after the neurons reinternalize the phosphatidylserine, the microglia do not phagocytose the neurons, and, therefore, the neurons remain alive [23,43]. Transient MCAO in rats induced phosphatidylserine exposure (as indicated by annexin V uptake) on live neurons, which peaked 3 days after stroke and largely reversed at 7 days [44]. The mechanism of this reversible phosphatidylserine exposure on neurons after ischemia was found to be calcium activation of the phosphatidylserine scramblase TMEM16F and the knockdown of the TMEM16F prevented microglial phagocytosis of stressed neurons in vitro and reduced functional deficits after transient MCAO in rats in vivo [45]. Thus, neurons can reversibly expose the eat-me signal phosphatidylserine via the activation of phosphatidylserine scramblases, and this makes these stressed, but viable, neurons potentially susceptible to phagocytosis by microglia, particularly in the presence of opsonins that bind to phosphatidylserine. Some of these phagocytic signals are depicted in Figure 2.

*Opsonins* are normally extracellular proteins, which, when bound to a cell, induce phagocytes to phagocytose that cell [35]. Phosphatidylserine exposed on neurons is recognized by the opsonin MFG-E8, which then drives the microglial phagocytosis of such phosphatidylserine-exposed neurons via the vitronectin receptors: integrins α_v_β_3_ or α_v_β_5_ [23,46]. MFG-E8 is upregulated after stroke, and the knockout of MFG-E8 prevents the delayed neuronal loss and long-term functional deficits after focal cerebral ischemia, indicating that blocking these opsonins can be beneficial [43]. Similarly, phosphatidylserine exposed on neurons can be recognized by the opsonin Gas6, which can then induce microglial phagocytosis of such neurons via the phagocytic receptor Mer tyrosine kinase (MerTK) [47]. Delayed neuronal loss and functional deficits after focal cerebral ischemia were reduced or eliminated in MerTK mutant rats [43]. This suggests that phosphatidylserine exposure drives microglial phagocytosis of stressed neurons after stroke and that blocking the phagocytic receptors on microglia responsible for this phagocytosis can be protective. 

Osteopontin can also act as an opsonin [48], is upregulated in infarct and peri-infarct regions after stroke [49,50], and binds to cellular debris and debris within microglia [49]. Osteopontin opsonises by binding integrin receptors α_v_β_1_, α_v_β_3_ and α_v_β_5_ on phagocytes [51] and possibly to calcium deposits on degenerating cells [50,52]. The knockout of the osteopontin gene (*Spp1*) reduces neuronal loss in a stroke model [50], consistent with osteopontin opsonising stressed/damaged neurons. However, ventricular injection of osteopontin reduced the secondary neurodegeneration of the thalamus after cortical stroke [53], suggesting a neuroprotective role, and see Section 8 below. 

Phosphatidylserine exposed on neurons can also bind the opsonin: complement component C1q, although C1q can bind a variety of other eat-me signals and opsonins [32,54]. C1q was greatly increased in or on neurons one day after MCAO in mice, and a C1q-blocking protein reduced subsequent brain damage [55]. An anti-inflammatory drug (salidroside) inhibited C1q expression in the brain 48 h after MCAO and reduced neuronal loss [56]. The knockout of C1q decreased the infarct volume 72 h after cerebral ischemia in neonatal mice [57,58]. However, the knockout of C1q did not change infarct volume 24 h after MCAO in adult mice [59,60]. 

In addition to acting as an opsonin itself, C1q binding to a cell can induce C3 complement activation, resulting in the deposition of the opsonins C3b, iC3b and C3d on neurons [60,61]. An inhibitor of C3 activation, Crry, targeted to ischemic neurons, prevented phagocytosis of stressed-but-salvageable neurons and synapses in the ischemic penumbra, and improved cognitive function in mouse models of stroke [61,62]. Stressed, but salvageable neurons, identified by c-fos expression, accumulated in the ischemic penumbra, and the phagocytosis of these stressed neurons by microglia was prevented by Crry or C3 knockout [61]. Microglia may also phagocytose synapses for at least 30 days after stroke, and blocking the complement-mediated microglial phagocytosis of synapses improved cognitive outcomes in a mouse model of stroke [62]. This suggests that complement contributes to microglial phagocytosis of neurons and synapses after stroke, and blocking this is beneficial for outcomes. Complement is known to mediate the microglial phagocytosis of synapses during development and neurodegeneration [26]; therefore, it has the potential to do this after stroke.

Microglial phagocytosis of neurons also normally requires the inflammatory activation of the microglia, resulting in microglial: (a) migration, (b) proliferation, (c) transition to amoeboid morphology, (d) the release of oxidants, cytokines and glutamate that stress neurons, (e) the release of opsonins, (f) expression of more phagocytic receptors and lysosomes and (g) expression of other factors stimulating phagocytosis. For example, the ganglioside GD3 was greatly increased on microglia (due to the upregulation of GD3 synthase) 2–7 days after transient global brain ischemia and increased the microglial phagocytosis of neurons, and GD3 synthase knockout mice had reduced microglial phagocytosis of neurons, reducing the delayed loss of neurons after ischemia [63]. This again suggests that factors increasing microglial phagocytosis can increase neuronal loss after ischemia while decreasing microglial phagocytosis can reduce neuronal loss.

Signaling between neurons and microglia after stroke may also inhibit phagocytosis. For example, microRNA-98 was expressed by penumbra neurons one day after ischemic stroke in rats, and the microRNA was transferred in vesicles to microglia, where it apparently downregulated the platelet-activating factor receptor-mediated microglial phagocytosis of stressed but viable neurons, thus reducing the delayed neuronal death [64]. However, on the third day after ischemic stroke in rats, expression of microRNA-98 was greatly reduced and microglia were observed to phagocytose stressed but viable neurons. However, this was prevented by the overexpression of microRNA-98 [64]. MicroRNA-98 is protected by blocking the microglial expression of the platelet-activating factor receptor, which could act as a phagocytic receptor for stressed but viable neurons [64].

All of the above suggests that phagocytic signaling can regulate the microglial phagocytosis of neurons and synapses after stroke and that blocking this signaling can in some cases be beneficial by preventing excessive microglial phagocytosis of neurons or synapses. Note, however, that some of this signaling is not exclusive to phagocytosis; therefore, it is not always possible to conclude that the neuronal loss is due to phagocytosis. For example, complement activation can induce phagocytosis but also the membrane attack complex, which might also contribute to neuronal damage after stroke.

## 5. Evidence for Microglial Phagocytosis Causing Neuronal Loss in Peri-Infarct Areas after Stroke

Selective neuronal loss may occur days after transient middle cerebral artery occlusion in rodent striatum (and to a lesser extent in the cortex) but may increase over weeks [4,7,8,9,10,11,12,13]. Evidence for microglial phagocytosis causing neuronal loss in peri-infarct areas after a stroke includes the following. Transient ischemia induced by endothelin 1 induced delayed loss of neurons in peri-infarct areas, accompanied by the microglial phagocytosis of neurons and the knockout of the opsonin MFG-E8 or phagocytic receptor MerTK, prevented delayed neuronal loss and long-term functional deficits [43]. The knockdown of TMEM16F, which mediates reversible phosphatidylserine exposure on neurons after ischemia, prevented microglial phagocytosis of stressed neurons and reduced motor deficits after transient MCAO in rats [45]. Overexpression of microRNA-98 reduced microglial phagocytosis of penumbral neurons after stroke in rats and mice [64]. C1q was greatly increased in or on neurons one day after MCAO in mice, and a C1q-blocking protein reduced subsequent brain damage [55]. An inhibitor of C3 activation, Crry, prevented phagocytosis of stressed-but-salvageable neurons and synapses in the ischemic penumbra and improved cognitive function in a mouse model of stroke [61,62]. The knockout of the opsonin osteopontin reduced the delayed neuronal loss in peri-infarct areas and increased motor recovery seven weeks following stroke [50]. All of this evidence indicates that blocking phagocytic signaling can reduce neuronal loss in peri-infarct areas after stroke. Importantly, this protection is not transient but can be sustained for weeks after stroke (Table 2).

## 6. Evidence for Microglial Phagocytosis in Secondary Neurodegeneration of the Thalamus after Stroke

Secondary neurodegeneration and neuroinflammation may occur in the thalamus weeks or months after a cortical infarct caused by stroke [2,3,14,67,68], or may occur in the midbrain secondary to infarcts in the striatum [16,17,18,69]. There may also be progressive loss of hippocampus neurons, volume and function, secondary to cortical infarcts and accompanied by neuroinflammation [15,68,70]. Secondary neurodegeneration after stroke can lead to cognitive and motor dysfunction that may progress to dementia [2,70]. The thalamus has multiple, reciprocal axonal connections with the cortex (and similarly, the midbrain is reciprocally connected to striatum), so the secondary neurodegeneration could be due to: (i) damaged or lost synapses and axons in cortex causing retrograde degeneration of thalamic neurons, (ii) damaged or lost neurons in cortex causing anterograde degeneration of their axons and synapses in the thalamus, and/or (iii) this local degeneration triggering local excitotoxicity and inflammatory processes [2]. However, it was more recently reported that stroke in the motor cortex could induce (14 days later) widespread loss of dopaminergic neurons in the midbrain without direct neuronal connections to the infarct [71]. The authors concluded that the secondary neuronal loss must be mediated by indirect neuronal connections, possibly via reduced inhibitory neurotransmission leading to excitotoxicity [71]. Note that excitotoxicity can kill neurons directly but can also stress neurons such that they are phagocytosed by microglia [43].

In stroke patients with cortical infarcts, there was increased microglial activation in the thalamus 2–24 months after stroke, as indicated by PET (positron emission tomography) imaging of microglial markers [72] and microglial activation correlated with MRI (magnetic resonance imaging) measures of neurodegeneration [73]. In mouse models of secondary neurodegeneration of thalamus after cortical stroke, there were increased markers of microglial activation (Iba-1, MHC-II) and microglial phagocytosis (CD68, CD11b, CD11c, Axl, ApoE) in the thalamus one month after the stoke at the time of neuronal loss [74,75]. During the secondary neurodegeneration of thalamus, 7–56 days after stroke in mouse motor cortex, neuronal loss correlated with microglial activation and with neuronal nuclei (NeuN+) found within microglia, i.e. microglia phagocytosed neuronal cell bodies [67]. Microglial activation was accompanied by an inhibition of directed process extension by microglia, probably due to P2Y_12_ receptor downregulation or relocalization, but phagocytosis was increased [67]. Inhibitors of cathepsin B (a lysosomal protease required for microglial phagocytosis) reduced secondary neuronal loss in the thalamus after cortical stroke [76], indicating a potential role of microglial phagocytosis, but cathepsin B may be involved in other processes. Knockout of the opsonin osteopontin reduced neuronal loss and motor deficits in another mouse model of secondary neurodegeneration [50], again suggesting a role of microglial phagocytosis in such delayed neuronal loss.

## 7. Evidence for Microglial Phagocytosis in Delayed Neuronal Loss after Transient, Global Brain Ischemia

There is also some evidence for microglial phagocytosis contributing to neuronal loss after transient, global brain ischemia. The ganglioside GD3 was greatly increased on microglia (due to upregulation of GD3 synthase) 2–7 days after transient global brain ischemia and increased microglial phagocytosis of neurons, and GD3 synthase knockout mice had reduced microglial phagocytosis of neurons and reduced delayed loss of neurons after ischemia [63]. Partial knockout of the P2Y_12_ receptor, required for microglial recruitment, prevented the loss of neurons in the CA1 hippocampus after transient, global brain ischemia [36]. Inhibition of the P2Y_12_ receptor by clopidogrel also prevented this microglial recruitment and neuronal loss [36], and this is potentially translatable to stroke as clopidogrel is currently used in some stroke patients to block platelet aggregation.

## 8. Evidence That Microglial Activation and Phagocytosis Are Beneficial after Stroke

Microglia can be beneficial after stroke by: inhibiting inflammation, releasing neuroprotective growth factors (such as BDNF), promoting angiogenesis, stimulating neurogenesis, increasing axonal regeneration and blood–brain barrier protection [25,34]. In addition, microglial phagocytosis specifically may be beneficial after stroke by: (i) removing debris that promotes neuroinflammation and hinders regeneration, and (ii) synaptic, dendritic and axonal remodeling by microglia to rebuild functional neuronal networks [25,34]. Thus, for example, inhibition of the P2Y_6_ receptor (by MRS2578), required for microglial phagocytosis of neurons [77], increased brain damage after transient MCAO in mice [33]. However, the specificity of MRS2578 and its ability to cross the BBB is unknown, and peripheral MRS2578 can cause hypotension [78], which could exacerbate ischemic brain damage. MRS2578 injected into the brain can prevent microglial phagocytosis of stressed neurons and loss of neurons induced by LPS/endotoxin [79]. 

Another example is saponin PF11, which reduced brain damage after MCAO, by increasing the microglial phagocytosis of myelin debris [80]. Similarly, STAT6-mediated M2 activation of microglia promoted the microglial phagocytosis of neuronal debris that was beneficial in a stroke model [81]. Knockout of the microglial phagocytic receptor TREM2 increased infarct size and functional deficits after stroke in mice, potentially by reducing phagocytosis [82,83]. Sphingosine-1-phosphate (S1P) may act as a ligand for TREM2, mediating the microglial phagocytosis of apoptotic neurons, and an S1P analog reduced infarct size and neurological deficits after stroke in mice, potentially by increasing phagocytosis [84]. Note, however, that S1P is also a find-me signal [40].

Intravenous injection of the opsonin MFG-E8 reduced infarct size and motor deficits 48 h after permanent MCAO in rats [85]. Similarly, infarct size was increased in MFG-E8 knockout mice and decreased by injection of MFG-E8, although this was attributed to MFG-E8 suppressing inflammation [86]. 

As noted in Section 4 above, osteopontin can act as an opsonin [48], and knockout of osteopontin reduced neuronal loss in a stroke model [50], consistent with osteopontin mediating excessive phagocytosis. However, ventricular injection of osteopontin reduced the secondary neurodegeneration of the thalamus after cortical stroke [53], consistent with osteopontin being neuroprotective. On the other hand, small peptides containing the RGD integrin-binding motif of osteopontin were protective in transient MCAO models [66,87], suggesting these peptides block integrin receptors that are mediating pathological phagocytosis or neuroinflammation.

Microglia may also phagocytose non-neuronal cells in the ischemic penumbra after stroke, including invading neutrophils, so that microglial depletion increased neutrophil numbers and enlarged the infarct, indicating that the microglial phagocytosis of activated neutrophils can be beneficial [88]. On the other hand, microglia in the stroke penumbra were found to phagocytose endothelial cells resulting in the breakdown of the blood–brain barrier, so that the depletion of microglia was beneficial, suggesting that the microglial phagocytosis of endothelial cells was detrimental [89].

## 9. Conclusions

**Figure 3:**
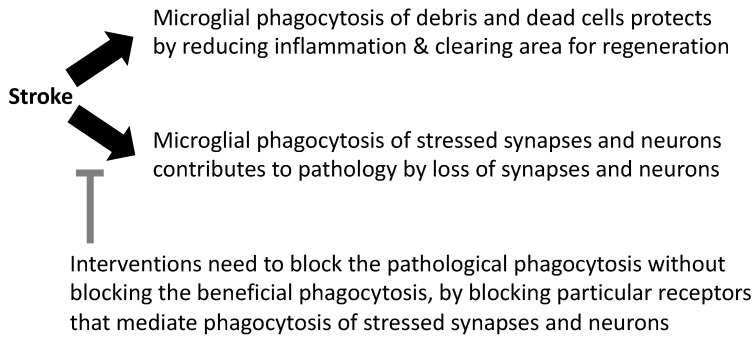
The divergent roles of microglial phagocytosis in stroke pathology.

There is evidence outlined above to suggest that microglial phagocytosis can be either protective or damaging after stroke (Figure 3). This may vary with the severity of the insult, as neurons subjected to severe insults are unlikely to be rescued by inhibiting phagocytosis, and severe insults generate debris that needs to be cleared and remodeled by microglial phagocytosis. Perhaps more importantly, the means by which microglial phagocytosis is inhibited may be crucial because different phagocytic receptors and opsonins bind and enable phagocytosis of different phagocytic targets, such as debris, myelin, synapses or stressed neurons, via different signaling molecules [35]. The age and sex of mice and patients can also affect the response to stroke and treatments, as microglial expression and responses vary with age and sex [90]. It will be important to test whether blocking specific microglial receptors is beneficial or detrimental in particular stroke models.

Potential targets to reduce delayed neuronal loss after stroke include: TMEM16F, which mediates the phosphatidylserine exposure and subsequent phagocytosis after stroke [45], and complement activation and subsequent phagocytosis via complement receptor 3 [55,60,61,62]. The C3a receptor [41,42,60] and the P2Y_12_ receptor [36], which both mediate microglial recruitment, are also potentially attractive targets to prevent neuronal loss after stroke. Blocking the phagocytic receptors MerTK and the vitronectin receptors (integrins α_v_β_3_ or α_v_β_5_) can also prevent the phagocytosis of stressed neurons in stroke models [23,43]. Recently it was shown that the knockout of the phagocytic receptor MerTK, specifically in microglia and macrophages, reduced synaptic loss, brain atrophy and neurological deficits in mouse models of both ischemic and hemorrhagic stroke, whereas the knockout of the phagocytic receptor MEGF10, specifically in astrocytes, reduced synaptic loss and brain atrophy only in the ischemic model of stroke [65]. The latter finding suggests that phagocytosis by both microglia and astrocytes can be detrimental in ischemic stroke. The phagocytic vitronectin receptor (α_v_β_3_) can be blocked with RGD-containing peptides, and the nasal delivery of these peptides provides substantial protection in rat models of ischemic stroke [66,87]. Hence, more research and clinical trials targeting microglial phagocytosis are likely to be of benefit in stroke medicine.

## Figures and Tables

**Table 1:** Published examples of delayed neuronal loss after stroke, indicating reference, stroke model used, site and time of neuronal loss.

Ref.	Stroke Model	Area of Neuron Loss	Time of Neuron Loss
[7]	15 min MCAO in rats	Striatum	0–16 weeks
[8]	30 min MCAO in mice	Striatum	0–6 weeks
[9]	60 min MCAO in rats	Striatum	0–6 weeks
[10]	20 min MCAO in rats	Striatum and cortex	1–7 days
[11]	20 min MCAO in rats	Striatum	1–7 days
[12]	15 min MCAO in hypertensive rats	Cortex	0–4 weeks
[13]	45 min MCAO in hypertensive rats	Cortex	0–4 weeks
[18]	30 min MCAO in mice	Cortex	Unknown
[19]	30 min MCAO in rats	Striatum	0–5 days
[6]	Transient 4 vessel occlusion in rats	Cortex and hippocampus	1–3 days
[15]	Photothrombosis in mouse cortex	Hippocampus	7–84 days
[5]	5 min carotid occlusion in gerbil	Hippocampal CA1	2–4 days
[3]	Ischemic stroke in human cortex	Thalamic atrophy by CT	0–12 month
[16]	Basal ganglia stroke in humans	Substantia nigra	Unknown
[17]	MCAO in humans	Substantia nigra	Unknown
[20]	Cardiac arrest in humans	Hippocampus	1–7 days

**Table 2:** Examples of interventions targeting microglial phagocytosis, which are of benefit in stroke models.

Ref.	Stroke Model	Intervention	Reduced Levels of
[43]	Transient, focal ischemia induced by endothelin 1 in mice	MFG-E8 knockout	Brain atrophy and motor deficits at 28 days
[43]	Transient, focal ischemia induced by endothelin 1 in rats	MerTK knockout	Brain atrophy and motor deficits at 28 days
[65]	Collagenase-induced intracerebral hemorrhage	MerTK knockout in microglia	Brain atrophy and motor deficits at 14 days
[65]	Transient MCAO in mice	MerTK knockout in microglia	Brain atrophy and motor deficits at 14 days
[66]	Transient MCAO in rats	RGD-peptides inhibiting α_v_β_3_	Infarct and motor deficits at 2 days
[45]	Transient MCAO in rats	TMEM16F knockdown	Neuron loss at 3 days Motor deficits 14 days
[64]	Transient MCAO in rats and mice	MicroRNA-98 overexpression	Neuron loss and neuro-deficits at unclear time
[55]	Transient MCAO in mice	C1q-blocking protein sCR1	Neuro-deficits 1 day, infarct at 3 days
[60]	Transient MCAO in mice	C3 knockout	Infarct and neurological deficits at 1 day
[60]	Transient MCAO in mice	C3a-receptor antagonist	Infarct and neurological deficits at 1 day
[61]	Transient MCAO in mice	C3 inhibitor Crry	Neuron loss and neurodeficits at 15 days
[62]	Embolic MCAO in mice	C3 inhibitor Crry	Synapse loss and cognitive deficits at 30 days
[50]	dMCAO + hypoxia model of 2ry neurodegeneration in mice	Osteopontin knockout	Motor deficits 14 days, neuron loss at 49 days
[63]	Transient global brain ischemia in mice	GD3 synthase knockout	Neuron loss at 4 days
[36]	Transient global brain ischemia in mice	P2Y12 knockout or inhibition	Neuron loss at 3 days

## Data Availability

Any data relevant to this manuscript can be requested by emailing the corresponding author.
